# Acute Renal Infarction as an Initial Manifestation of Factor V Leiden Mutation: A Case Report

**DOI:** 10.7759/cureus.99975

**Published:** 2025-12-23

**Authors:** Bassem Derbas, Nadia Katrib, Violette E Issa, Jessika Eid

**Affiliations:** 1 Faculty of Medicine, University of Balamand, Koura, LBN; 2 Internal Medicine, Faculty of Medicine, Lebanese University, Beirut, LBN; 3 Nephrology, Nini Hospital, Tripoli, LBN

**Keywords:** factor v leiden, hypercoagulability, ischemic kidney injury, renal infarction, thrombophilia

## Abstract

Factor V Leiden is one of the most prevalent forms of inherited thrombophilia. While its usual presentation is a deep vein thrombosis and pulmonary embolism, it can manifest less commonly as a renal infarction. Here, we describe a case of a previously healthy woman who was found to have a renal infarction triggered by a hypercoagulable state associated with the Factor V Leiden mutation. This case highlights the importance of considering thrombophilia as a differential diagnosis in cases of insidious acute renal infarction, especially in patients with no identified risk factors.

## Introduction

Renal infarction is an uncommon occurrence characterized by the sudden interruption of blood flow through the renal artery or its segmental branches, leading to ischemia of the affected kidney parenchyma. Etiologies of this condition involve either thromboembolism or in situ thrombosis [1]. Thromboembolism is usually of cardiac origin, while in situ thrombosis is the result of a hypercoagulable state, an injury to or dissection of the renal artery [1].

One notable cause of a hypercoagulable state is the factor V Leiden mutation, which causes resistance to activated protein C. It is inherited in an autosomal dominant manner. Heterozygosity of factor V Leiden is the most common inherited abnormality in patients of the white race with hypercoagulability issues [2]. Factor V Leiden results from a single nucleotide change in the F5 gene that produces the Arg506Gln substitution. This mutation renders factor V resistant to activated protein C and promotes a prothrombotic state. This variant is most commonly present in individuals of European descent and represents the most prevalent inherited thrombophilia in this population, with approximately 3% to 8% carrying the heterozygous form of the gene mutation. In contrast, it is less prevalent in East Asian, Indigenous American, and most sub-Saharan African groups, and appears in only about 1%-2% of African Americans due to partial European admixture. Factor V Leiden has been identified in up to one-fifth of Caucasian patients with venous thromboembolism, highlighting its role as a major contributor to the hypercoagulable state in this population.

Renal infarction presents acutely with nonspecific symptoms, most commonly unilateral flank pain mimicking renal colic, nephrolithiasis, or pyelonephritis, which makes its diagnosis challenging. Other associated symptoms include fever, nausea, new onset or worsening hypertension, and hematuria, which can be microscopic or gross. Imaging plays a pivotal role in the diagnosis of renal infarction, with contrast-enhanced computed tomography (CT) being the gold standard, showing a wedge-shaped non-enhancing region of the renal parenchyma and most specifically the cortical rim sign [3]. In cases where patients have associated acute kidney injury, renal failure, or known contrast allergy, contrast-enhanced ultrasound (CEUS) appears to be an excellent alternative for contrast-enhanced CT.

## Case presentation

**Figure 1:**
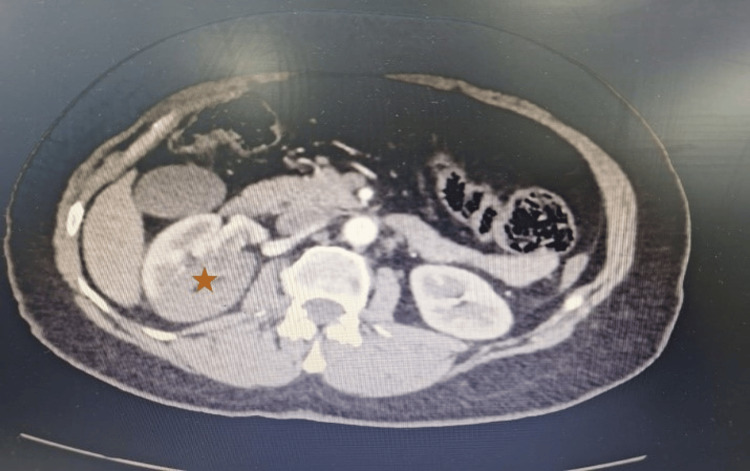
CT of the abdomen and pelvis with intravenous contrast demonstrating a wedge-shaped area of hypoenhancement with abrupt occlusion of the posterior segmental artery and a positive cortical rim sign, consistent with acute renal infarction (indicated by an asterisk).

A 58-year-old female, known to have dyslipidemia on rosuvastatin 10 mg daily, a non-smoker with no known allergies, presented to the emergency department for an acute onset of severe right flank pain radiating to the right groin of a few hours' duration associated with nausea and four episodes of vomiting. She denies having fever, chills, dysuria, or hematuria. Her vital signs were stable. Her physical exam showed right costovertebral angle tenderness. Laboratory findings on presentation showed borderline white blood cell (WBC) count of 10,600/mm³ (reference range: 4,400-11,300/mm³) with left shift (neutrophils at 78%), normal creatinine level of 0.7 mg/dL, negative C-reactive protein (CRP) of 1.4 mg/L (reference range: 0-5 mg/L), mild acidosis (CO_2_ 21 mmol/L) and a normal urine analysis (WBC 6-10/HPF, RBC 0-2/HPF, no nitrites, no proteins, no blood). The patient was in severe right flank pain radiating to the right groin area, refractory to analgesics. Further evaluation by CT scan of the abdomen and pelvis with intravenous contrast revealed a wedge-shaped area of hypoenhancement with abrupt occlusion of the posterior segmental artery, along with a positive cortical rim sign. These features were suggestive of acute renal infarction. The main right renal artery and the anterior segmental branch were permeable. There was no perinephric infiltration. Normal excretion of the right kidney and normal pelvi-calyceal morphology were noted. Biliary lesions appeared in segment V of the liver (Figure 1).

Further questioning following the diagnosis of renal infarct did not show that the patient had any episode of deep venous thrombosis, pulmonary embolism, or spontaneous abortions.

**Figure 2:**
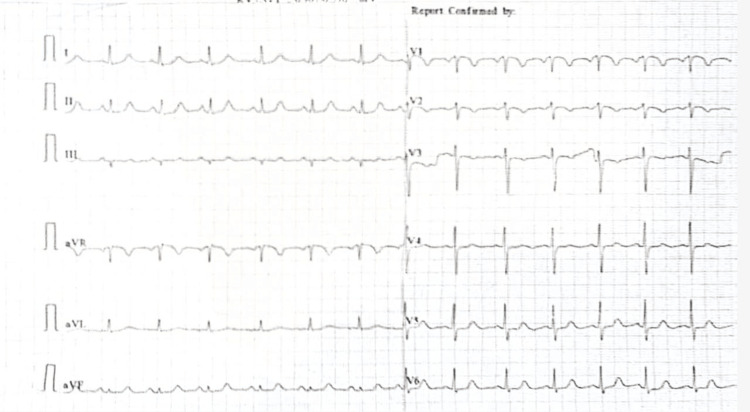
Electrocardiogram (ECG) showing normal sinus rhythm.

The patient was admitted for a renal infarct of unknown etiology and was started on symptomatic treatment with intravenous morphine using a syringe pump. Further investigations were conducted to determine the underlying cause, including cardiac evaluation to rule out thromboembolic sources and a hypercoagulable workup to assess for in situ thrombus. The cardiology workup revealed a sinus rhythm on the electrocardiogram (ECG) (Figure 2). Further evaluation by transthoracic and transesophageal echocardiography showed no features suggestive of atrial fibrillation (absence of left atrial dilation or appendage thrombus) or bacterial endocarditis (absence of valvular vegetations), eliminating these as potential embolic sources. Moreover, an extensive workup was done to rule out vasculitis or other autoimmune diseases as possible causes of the infarct, but the results were negative (negative antiphospholipid IgM and IgG, negative antinuclear antibody panel, negative ANCA (antineutrophil cytoplasmic antibodies), and normal C3 and C4 levels).

On the other hand, blood was drawn to check for any mutation responsible for this hypercoagulable state. After withdrawal of blood, therapeutic low molecular weight heparin (60 mg subcutaneously every 12 hours) was initiated. Hematological workup revealed a heterozygous mutation of the factor V Leiden. The results are summarized in Table 1.

**Table 1:** Hematological workup results. IgM, immunoglobulin M; IgG, immunoglobulin G

Hypercoagulable workup	Result	Reference values
Protein C	130%	80%-130%
Protein S	99%	60%-130%
Antiphospholipid IgM	1.4 MPL-U/mL	<10 MPL-U/mL
Antiphospholipid IgG	1.8 GPL-U/mL	<10 GPL-U/mL
Homocysteine	12.92 micromol/L	5.46-16.2 micromol/L
Prothrombin	Normal	
MTHFR 677	Normal	
MTHFR 1298	Heterozygous mutation	
Factor V Leiden	Heterozygous mutation	

Two days after admission, she developed a fever that persisted for four days. Repeated urinalysis was normal. Hemocultures were withdrawn twice. Both urine cultures (obtained on admission and during the febrile episode) and blood cultures showed no growth of pathogenic organisms. During this period, inflammatory markers increased, reaching a peak white blood cell count of 13,100/µL with 81% neutrophils and a CRP level of 318.3 mg/L on the fifth day of admission. Procalcitonin was negative. Fever was deemed to be due to renal necrosis. Notably, throughout hospitalization, the patient’s serum creatinine remained within the normal range, with no evidence of acute kidney injury despite segmental ischemia of the right kidney.

Once clinical and hemodynamic stability were achieved, the patient was discharged home on therapeutic anticoagulation with apixaban (5 mg every 12 hours for six months) and scheduled for follow-up with a hematologist. Given the autosomal dominant inheritance pattern, screening of the patient’s siblings and offspring was recommended to identify potential carriers. Both parents are deceased; therefore, their carrier status could not be determined.

## Discussion

Renal infarction results from occlusion of the main renal artery or its segmental branches [1]. It typically presents as severe flank or abdominal pain, sometimes accompanied by impaired renal function. Hematuria or proteinuria may be present, along with elevated lactate dehydrogenase (LDH) levels [1]. To diagnose renal infarction, a non-contrast abdominal CT scan is usually performed. If no changes are noted despite high suspicion, a contrast-enhanced CT scan will be the next best step in confirming the infarction [1]. Most commonly, renal infarction presents as wedge-shaped focal infarcts, but it can also manifest as global or multifocal infarcts of the kidneys [3].

Renal infarctions usually arise from emboli stemming from cardiac or other systemic sources [4]. In this patient, thorough investigations to determine the etiology of the infarct failed to determine any cardiac or aortic embolic origin, atrial fibrillation, vasculitis, or other hypercoagulable state such as antiphospholipid syndrome or malignancy [4]. Moreover, the patient had no history of recent surgery or trauma that could explain this thrombotic state [5]. In the light of the absence of these conditions, inherited thrombophilia should be investigated as a possible etiology. In this patient, genetic testing revealed a heterozygous mutation of factor V Leiden, which confers resistance to activated protein C, a natural anticoagulant. It is important to note that even though this patient also had a heterozygous mutation in the MTHFR 1298 gene, the latter cannot induce a hypercoagulable state by itself, especially since the homocysteine level was within normal range. Hence, we can conclude that the infarction was due to factor V Leiden. The solo manifestation of this mutation as a renal infarct is rare, especially in this *grand multipara* patient with no prior history of abortions, which are usually expected in women with a hypercoagulable state [6].

The likelihood of renal recovery depends on several factors, mainly the underlying risk factors and the duration between symptom onset and treatment initiation. Therefore, early diagnosis and treatment are crucial to optimize renal function recovery and prevent recurrence of other thrombotic events. Treatment is individualized based on one’s risk factors. Our patient was treated with therapeutic low-molecular-weight heparin (60 mg subcutaneous injection every 12 hours) and discharged on oral anticoagulant for six months.

## Conclusions

Renal infarction, although rare, can be one of the manifestations of the factor V Leiden mutation. Therefore, physicians should consider thrombophilia as a possible etiology in the setting of unexplained renal infarction. Treatment should be tailored according to each patient’s genetic profile and potential factors that may influence their thrombotic risk.
